# Overexpression of *PIK3R1* promotes hepatocellular carcinoma progression

**DOI:** 10.1186/s40659-018-0202-7

**Published:** 2018-11-29

**Authors:** Xuejun Ai, Lei Xiang, Zhi Huang, Shi Zhou, Shuai Zhang, Tao Zhang, Tianpeng Jiang

**Affiliations:** 1 The Key Laboratory of Medical Molecular Biology, Guizhou Medical University, Guiyang, 550002 China; 2 Department of Digestive, Guiyang First People’s Hospital, Guiyang, 550001 China; 3 Department of Interventional Radiology, Affiliated Hospital of Guizhou Medical University, Guiyang, 550002 China

**Keywords:** Hepatocellular carcinoma, PIK3R1, Apoptosis, Targeted therapy

## Abstract

**Background:**

Phosphoinositide-3-kinase, regulatory subunit 1 (PIK3R1) could regulate cancer cell proliferation important for cancer cell proliferation; however, its role in Hepatocellular carcinoma (HCC) remains largely unknown. Here, we investigated the role of PIK3R1 in HCC and examined the underlying molecular mechanisms.

**Methods:**

The expression of PIK3R1 was evaluated by immunohistochemistry and qRT-PCR in a series of HCC tissues. The mRNA and protein expression of PIK3R1 was used by qRT-PCR and western blot assays in a series of human HCC cell lines, and then we choose MHCC97H and HCCLM3 cells as a model to investigate the effect of PIK3R1 on HCC progression. The effects of PIK3R1 knowdown on cell proliferation, migration, apoptosis of HCC were assessed by the MTT assay, clonogenic assays, wound healing assay and flow cytometry in vitro. Western blot assay was performed to assess the expression changes of PI3K/AKT/mTOR signaling pathway.

**Results:**

Our results found that PIK3R1 was highly expressed in HCC tissues compared with adjacent normal tissues. Knockdown of PIK3R1 inhibited the proliferation, migration and promoted apoptosis of HCC cell lines. In addition, we proved that knockdown of PIK3R1 downregulated p-PI3K, p-AKT, and p-mTOR expressions in MHCC97H and HCCLM3 cells.

**Conclusions:**

In conclusion, PIK3R1 providing potential novel targets for the treatment of HCC.

## Background

Hepatocellular carcinoma (HCC) is the fifth most common cancer worldwide, and one of the leading cause of death among malignancies in China [1, 2]. Despite advances in diagnostic and treatment modalities, the prognosis for HCC has not significantly improved, and the 5-year survival rate for patients with HCC remains poor, which is largely attributable to the high rates of distant metastasis [3]. Thus, there is an urgent need to develop new strategies to HCC treatment.

Phosphoinositide 3-kinase (PI3K) is a heterodimer that consists of an SH2-containing regulatory subunit (p85) and a catalytic subunit (p110), with both subunits expressed in multiple isoforms [4]. There are eight isoforms of the regulatory subunit encoded by three different genes, Pik3R1, PIk3R2, and PIk3R3. In most eukaryotic cells, the gene products of PIk3R1 constitute 65–75% of the intracellular pool of regulatory subunits in the form of p85α [5]. It has been reported that deletion of PIk3R1 in liver may result in a marked reduction in insulin-stimulated PI3K activity with significant defection in glucose and lipid homeostasis, as well as in hepatic size and function [6]. An increasing number of PIK3R1 have been identified to be differentially expressed in many human cancers and implicated in tumor progression and metastasis [7]. Aberrations of PIK3R1 occur in endometrial cancers (EC), breast cancer, colon cancer, and glioblastomas, as an important therapeutic target through inhibiting mTOR (http://www.sanger.ac.uk/genetics/CGP/cosmic) [8]. However, the role of PIK3R1 in hepatocellular carcinogenesis remains unknown. In this study, we investigated the expression of PIK3R1 in human HCC by using qRT-PCR, western blot, and immunohistochemistry, and explored the potential role of PIK3R1 in HCC progression. In addition, we explored the possible mechanism PIK3R1 in human HCC.

## Methods

### HCC tissue samples

From 2005 to 2014, tumor samples and corresponding adjacent normal tissues were collected from 92 HCC patients receiving surgery at the Affiliated Baiyun Hospital of Guizhou Medical University. The corresponding adjacent normal tissue samples were obtained > 5 cm from the edge of the cancerous region and there were no obvious tumor cells evaluated by pathologist. These tissue samples were conserved in liquid nitrogen after collection or prepared in paraffin sections. No systemic or local treatment had been received before operation. Both tumor and nontumor tissues were histologically confirmed. All the tissue samples were obtained with informed consent from all the patients. This study was approved by the Institute Research Ethics Committee of Guizhou Medical University.

### Cell lines

HCC cell lines MHCC97L, Huh7, HepG2, HCCLM3, SMMC-7721, MHCC97H and normal liver cell lines HL-7702 were from the tumor cell bank of Chinese Academy of Sciences. All the cell lines were grown in Dulbecco’s modified eagle medium supplemented with 10% fetal bovine serum, 100 Ag/AL streptomycin, and 100 Ag/AL penicillin (pH 7.2–7.4) in a humidified incubator containing 5% CO_2_ at 37 °C.

### Immunohistochemistry

For each patient sample, three paraffin sections of 5 μm were prepared, for immunohistochemical staining. Sections were dewaxed using xylene, followed by hydration with ethanol solutions and addition of EDTA for antigen retrieval. Later, sections were blocked with normal goat serum for 30 min to eliminate non-specific binding. Sections were incubated with primary antibody against PIK3R1 (Abcam, Cambridge, UK). Sections were then incubated with biotin-labeled secondary antibodies for 30 min at room temperature, followed by staining with diaminobenzidine (DAB).

### Reverse transcription-quantitative PCR

Total RNA of tissues or cultured cells was isolated by using TRIzol reagent (Invitrogen). Total RNA (1 μg) was transcribed into cDNA by using a First-strand cDNA Synthesis System (Invitrogen). 1 μl DNA template was used to amplify by using Power SYBR^®^ Green PCR Master Mix (ABI, USA) on the 7500 real time PCR system (ABI, life technology). The reaction system was performed in a volume of 20 μl. The GAPDH was used as a loading control for each specific gene. Each experiment was performed three times and each sample was tested in triplicate. The sequences of human PIK3R1 primers were 5′-TAGCTCGCGCGATCTAGGGGC-3′ (sense) and 5′-CGCGATCAATAAAGCTAG-3′ (antisense). The primers for human GAPDH were 5′-GCACCGTCAAGGCTGAGAAC-3′ (sense) and 5′-TGGTGAAGACGCCAGTGGA-3′ (antisense).

### Western blot analysis

Whole cells were lysed on ice in a lysis buffer (RIPA, Beyotime, Shanghai, China) with a protease inhibitor mixture cocktail (Roche, Switzerland). After centrifugation at 12,000 rpm for 30 min at 4 °C, the protein concentrations of supernatants in samples were measured by the BCA protein assay (Thermo scientific, Rockford, IL, USA). Equal amounts of protein (30 μg) were separated by 10–12% NUPAGE Bis–Tris Gel (Invitrogen, CA, USA) electrophoresis (constant voltage: 120 mv) and transferred onto polyvinylidene fluoride (PVDF, 0.45 μm) membranes (constant current: 350 mA for 70/120 min). After being blocked by Tris-buffered saline and Tween 20 (TBST) buffer containing 5% non-fat powder milk for 2 h, the membranes were incubated with primary antibodies overnight on ice. After washing the membranes several times in TBST while agitating, detection was performed using the appropriate secondary HRP-conjugated anti-mouse or antirabbit antibody. Immunoreactive bands on the blots were visualized with enhanced chemiluminescence reagent ECL kit (Beit Haemek, Israel).

### Small interfering RNA transfection of human HCC cell line

Human PIK3R1 siRNA (5′-CCTAGCGCATATCGCC-3′) and control-siRNA were synthesized by GenePharma (shanghai, china). Cells were transfected with sh PIK3R1 or control-shRNA using Lipofectamine 2000 (Invitrogen, Life Technologies), according to the manufacturer’s instructions.

### MTT assays

The proliferation of cells was evaluated by the MTT assay. Cells were plated in a 96-well plate at 3 × 10^3^ cells/well and were allowed to grow for different times. The growth rate was determined by the cell number and was counted in triplicate every day by MTT assay. Briefly, cells were incubated with 50 μl of 0.2% MTT for 4 h at 37 °C in a 5% CO_2_ incubator. Following MTT incubation, 150 μl of 100% DMSO was added to dissolve the crystals. Viable cells were counted every day by reading the absorbance at 490 nm using a 96-plate reader BP800 (Dynex Technologies).

### Clone formation assay

Clone formation assay was conducted to examine the effect of PIK3R1-siRNA on cell growth in HCC cell lines. 4 × 10^5^ cells were plated in a 6-well plate. After 24 h of transfection, the cells were trypsinized, and 1000 single viable cells were plated in three 6-well plates. The cells were then incubated for 14 days at 37 °C in the condition of 5% CO_2_. Colonies were stained with 0.1% crystal violet, washed with water, and counted ten random fields manually. The colonies containing at least 100 cells were scored. The surviving fraction in PIK3R1-siRNA transfected cells was normalized to untreated control cells with respect to clonogenic efficiency.

### Wound healing assay

Wound healing assay was adopted to test the migration ability of HCC cells. In our study, cells were digested after transfection by specific siRNA and control siRNA to human PIK3R1 for 24 h in 6-well plates, 2 × 10^5^ cells were plated in 24-well plates, when cell confluence reached approximately 100%, the old medium was removed and the monolayer was wounded by scratching with a 10-μl sterile pipette tip lengthwise along the chamber, then cells were washed three times with PBS and cultured with serum-free medium at 37 °C. Images of migrating cells into the wound were photographed at 0 h and 48 h using an inverted microscope. The scratch width of the cells was confirmed by detecting the width of the monolayer wound at 0 and 48 h, and the migration index was counted as follows: migration index = (0 h scratch width − 48 h scratch width)/0 h scratch width) × 100 [9].

### Transwell migration assay

Cell migration ability was determined by transwell assays. The treated MHCC97H and HCCLM3 cells (1.0 × 10^5^/ml) were seeded in the upper chambers (BD Biosciences, NY, USA). The upper chamber was filled with serum-free medium and the lower chamber was supplemented with 10% fetal bovine serum. Hence this allowed the cells in the upper chamber migrate into the lower chamber. After 48 h incubation, the cells that had invaded through the membrane were stained with 0.1% crystal violet solution. The sections were observed by using a light microscope (magnification at ×100).

### Apoptosis assay

The apoptosis ability was measured by using Annexin V-FITC/PI apoptosis detection kit (BestBio, Shanghai, China). Cells were digested after transfection by specific siRNA and control siRNA to human PIK3R1 were washed with ice-cold PBS. The treated cells (1 × 10^6^ cells/ml) were suspended with 100 μl 1 × binding buffer and double stained with Annexin V-FITC/PI for 15 min according to the manufacturer’s instructions. Intensities of fluorescence signals were measured on a FACS Calibur flow cytometer (Becton-Dickinson, Franklin-Lakes, NJ, USA). The image of apoptosis was divided into four quadrants: all living cells (double negative), early apoptotic cells (Annexin V-positive, propidium iodide-negative), necrotic cells (Annexin V-negative, propidium iodide-negative positive), as well as late apoptotic cells (double positive). We counted the early apoptotic cells and the late apoptotic cells.

### Statistical analysis

For continuous variables, data are expressed as mean ± standard deviation (SD). The difference between PIK3R1 mRNA or protein expression in tumor tissue and that in adjacent normal tissues was evaluated using Student’s t-test. GraphPad Prism 5.0 Software was employed to perform statistical analysis. All statistical tests were two-tailed and statistical significance was assumed for p < 0.05.

## Results

### PIK3R1 expression levels are significantly upregulated in human HCC tissue and HCC cell lines of high metastatic potential

**Fig. 1:**
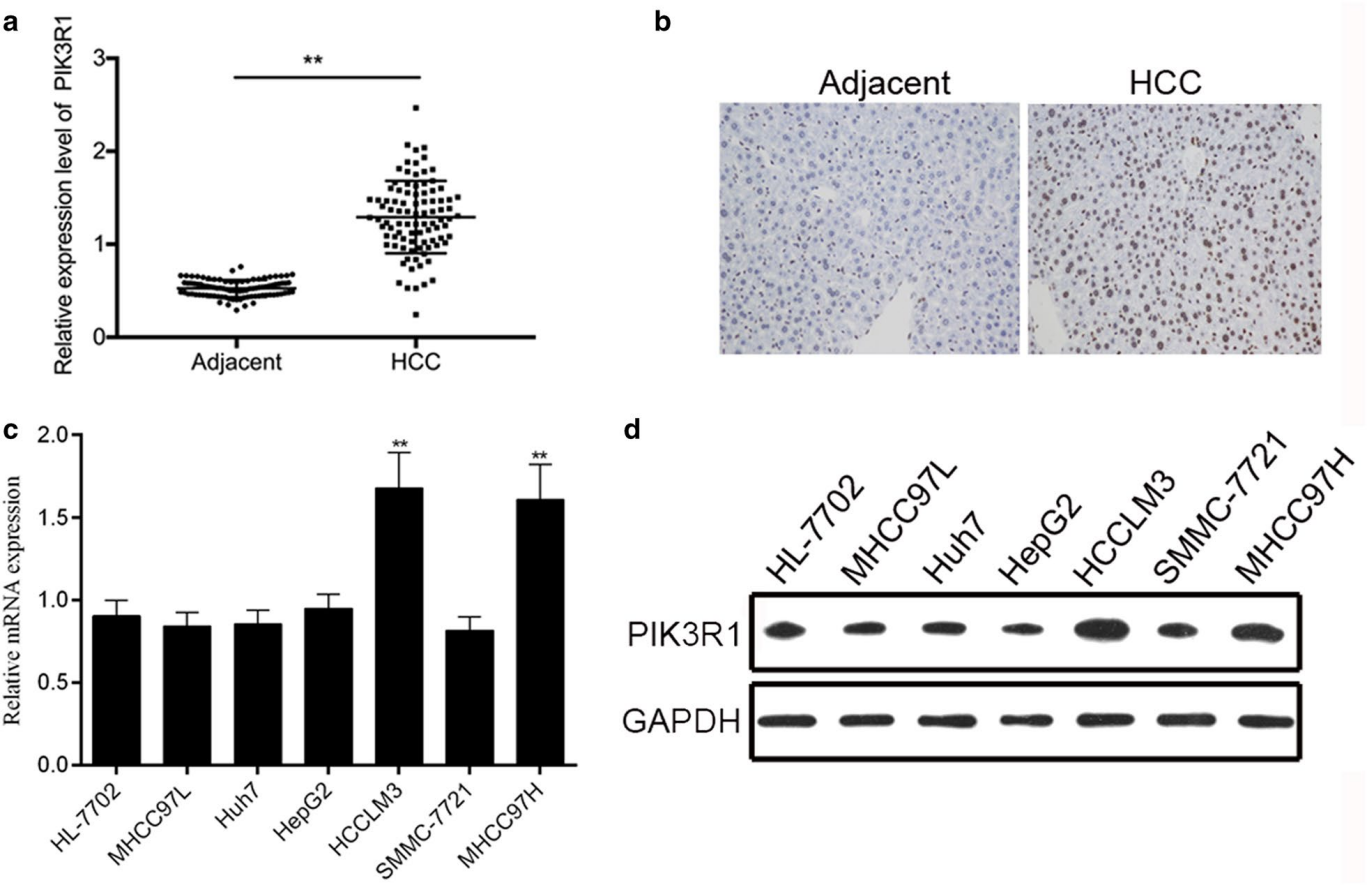
PIK3R1 expression levels are significantly upregulated in human HCC tissue and HCC cell lines of high metastatic potential. **a** PIK3R1 mRNA expression levels in 92 paired HCC tissues and corresponding nonneoplastic liver tissues expressed as relative expression normalised to the expression of GAPDH; **p < 0.01, **b** immunohistochemical staining of PIK3R1 in HCC tissues. (Original magnification, ×200); **c** PIK3R1 mRNA expression levels in a series of human HCC cell lines; **p < 0.01, versus the HL-7702 cell. **d** PIK3R15 protein expression in a series of human HCC cell lines

qRT-PCR was performed to detect the expression of PIK3R1 mRNA in 92 paired HCC tissues and corresponding adjacent tissues. PIK3R1 expression was significantly high in HCC tissues compared with the related normal pericarcinomatous tissues (Fig. 1a). In addition, statistical results showed that PIK3R1 expression was closely associated with lymphatic metastasis (p = 0.029), distal metastasis (p = 0.004) and pathologic tumor, node, metastasis stage (TNM stage, p = 0.002), but not age (p = 0.548), gender (p = 0.484) and tumor size (p = 0.503, Table 1). Immunohistochemical staining results showed that PIK3R1 expression in HCC specimens was significantly upregulated compare with adjacent non-tumoral liver tissue (Fig. 1b). Then, we detected the mRNA and protein expression of PIK3R1 in a series of human HCC cell lines, including MHCC97L, Huh7, HepG2, HCCLM3, SMMC-7721 and MHCC97H by qRT-PCR and Western Blot analyses, respectively. Our results showed that HCCLM3 and MHCC97H cells (high metastatic potential) showed the higher expression of PIK3R1 (Fig. 1c, d). Thus, we used MHCC97H and HCCLM3 cells as a model to investigate the effect of PIK3R1 on HCC progression.

**Table 1:** Correlations between PIK3R1 expression and clinicopathologic characteristics in HCC cancer

Clinicopathologic Characteristics	No. of patients	PIK3R1		*p* value
		High	Low	
Age (year)				
> 60	63	34 (54.0%)	29 (46.0%)	0.548
≤ 60	29	16 (55.2%)	13 (44.8%)	
Gender				
Male	57	26 (45.6%)	31 (54.4%)	0.484
Female	35	15 (42.9%)	20 (57.1%)	
Tumor size (cm)				
< 3	38	21 (55.3%)	17 (44.7%)	0.503
≥ 3	54	31 (57.4%)	23 (42.6%)	
Lymphatic metastasis				
N0	59	24 (40.7%)	35 (59.3%)	0.029*
N1–N4	33	21 (63.6%)	12 (36.4%)	
Distal metastasis				
M0	79	21 (26.6%)	58 (73.4%)	0.004**
M1	13	9 (69.2%)	4 (30.8%)	
TNM stage				
0 & I & II	64	19 (29.7%)	45 (70.3%)	0.002**
III & IV	28	18 (64.3%)	10 (35.7%)	

*TNM stage* pathologic tumor, node, metastasis stage

*p < 0.05, **p < 0.01

### PIK3R1 associated directly with the ability of cell proliferation of HCC cell lines

**Fig. 2:**
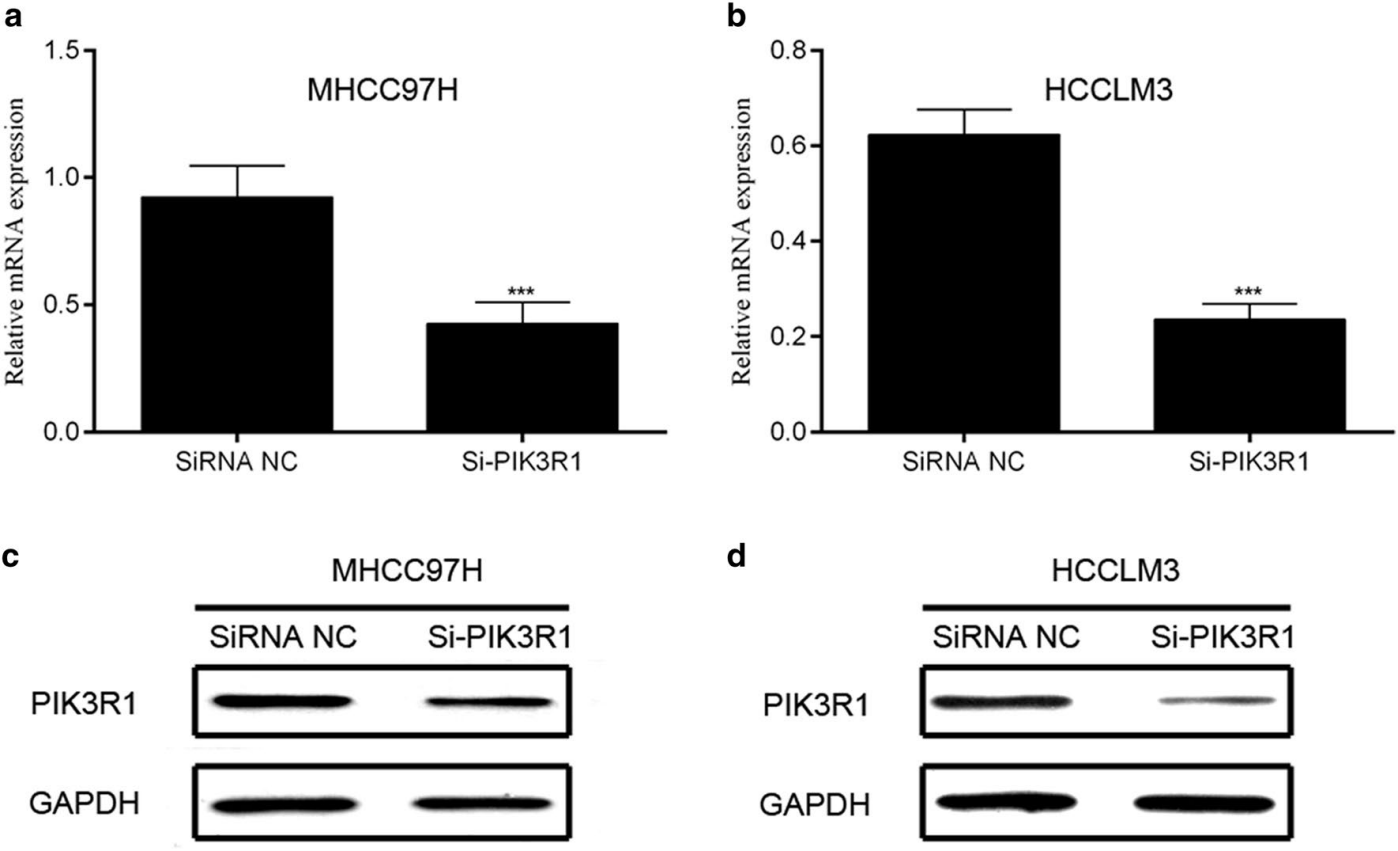
MHCC97H and HCCLM3 cells were infected with PIK3R1siRNA or siRNA NC. **a**, **b** PIK3R1 mRNA expression was analyzed by qRT-PCR; **c**, **d** MHCC97H and HCCLM3 cells were infected with PIK3R1 siRNA or siRNA NC. PIK3R1 protein expression was analyzed by western blotting. ***p < 0.001

To further assess the biological function of PIK3R1 in HCC, we used the siRNA to knockdown the PIK3R1 level. As shown in Fig. 2, PIK3R1 expression was distinctly decreased at mRNA and protein levels in MHCC97H and HCCLM3 compared with siRNA NC group, indicating that the specific siRNA of PIK3R1 effectively suppressed the expression of PIK3R1 in HCC cell lines.

**Fig. 3:**
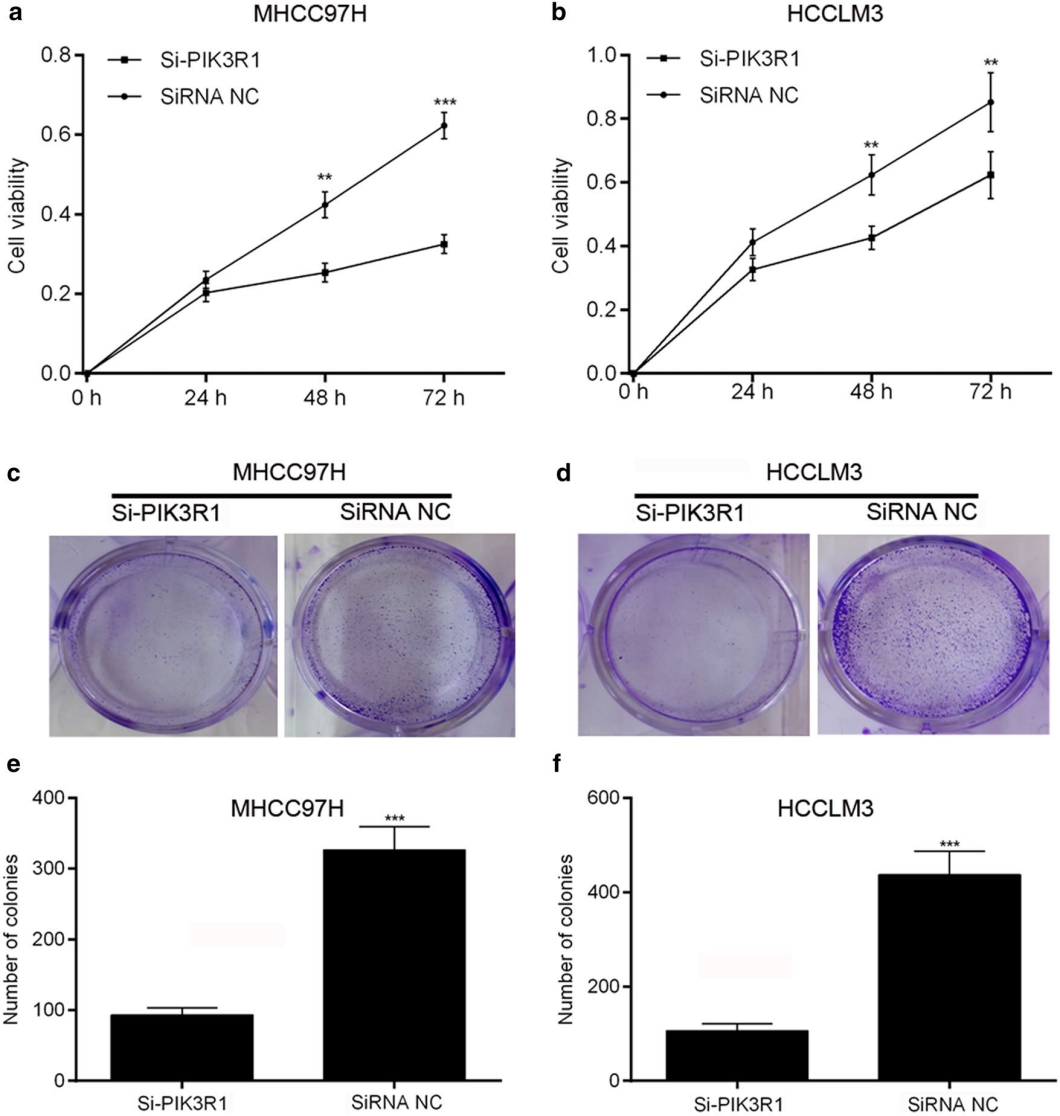
PIK3R1 knockdown inhibits the proliferation of MHCC97H and HCCLM3 cells. **a**, **b** Cell proliferation was measured by MTT assay; **c**–**e** colony formation was analyzed by colony-formation assay and quantification of colonies number. **p < 0.01, ***p < 0.001

To further test whether PIK3R1 were related to proliferation ability of HCC cells, we measured the effects of PIK3R1 expression levels on cancer cell proferation by MTT and Clonogenic assays. As shown in Fig. 3a, b, PIK3R1 knockdown was associated with significantly decreased cell viability of HCCLM3 and MHCC97H cells. Furthermore, PIK3R1 knockdown in HCCLM3 and MHCC97H cells consistently reduced the colony formation ability (Fig. 3c–f), suggesting that PIK3R1 may act as an oncogene involved in the promotion of HCC cell proliferation.

**Fig. 4:**
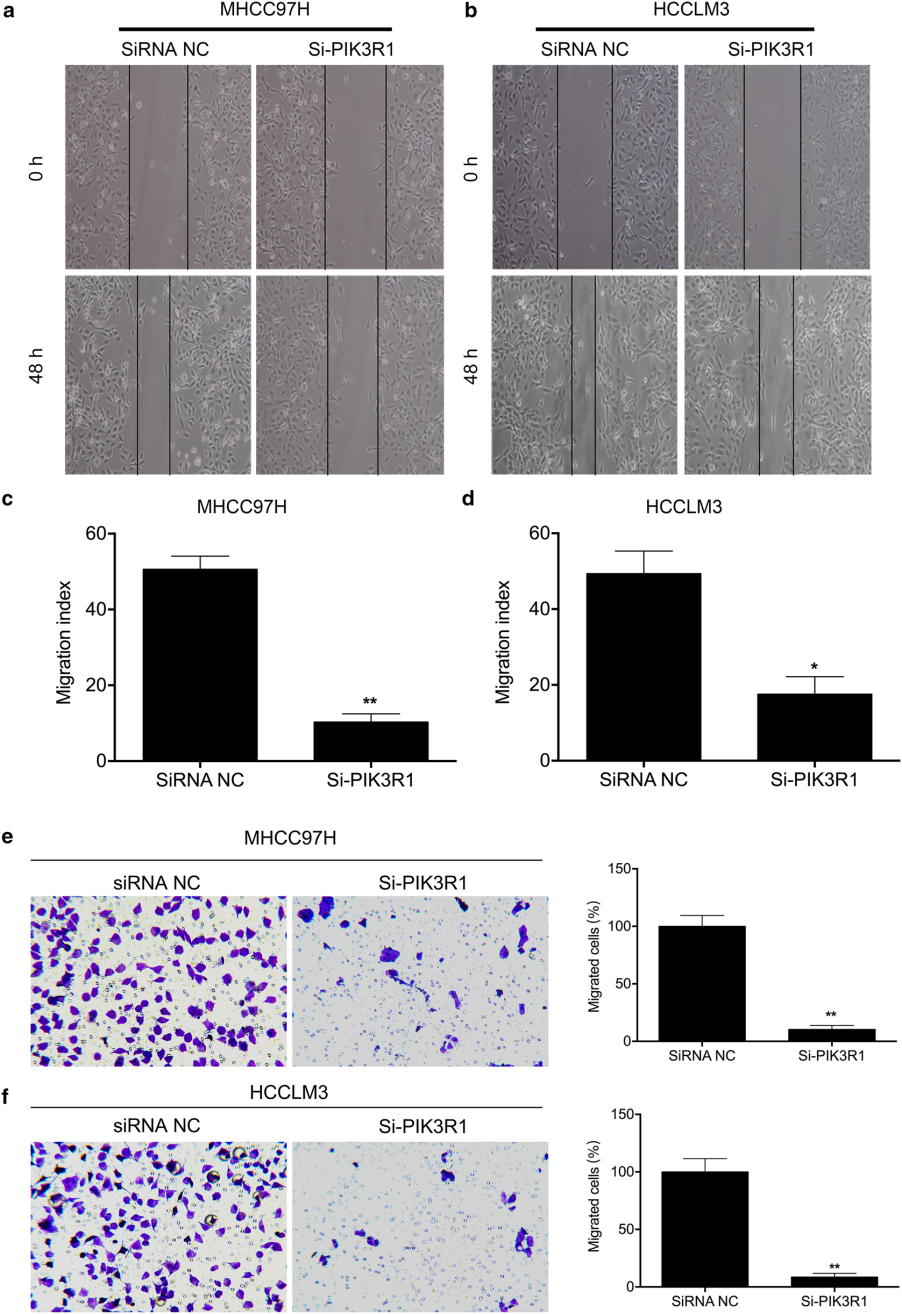
PIK3R1 knockdown inhibits the migration and invasion of MHCC97H and HCCLM3 cells. **a**, **b** Cell migration was measured by wound healing assays; **c**, **d** quantification of migration index. **e**, **f** Cell migration ability was measured by transwell assay in MHCC97H and HCCLM3 cells after PIK3R1 knockdown. The migrated cells were calculated. **p < 0.01 vs. siRNA NC group

Next, we carried out scratch wound-healing and transwell assay to evaluated whether PIK3R1 regulated the ability of migration of HCC cells. We found that knowdown of PIK3R1 markedly diminished wound-healing capacity and decreased the migrated cells (Fig. 4), suggesting that PIK3R1 promotes migration by HCC cells in vitro.

### Downregulation of PIK3R1 expression increases cell apoptosis

**Fig. 5:**
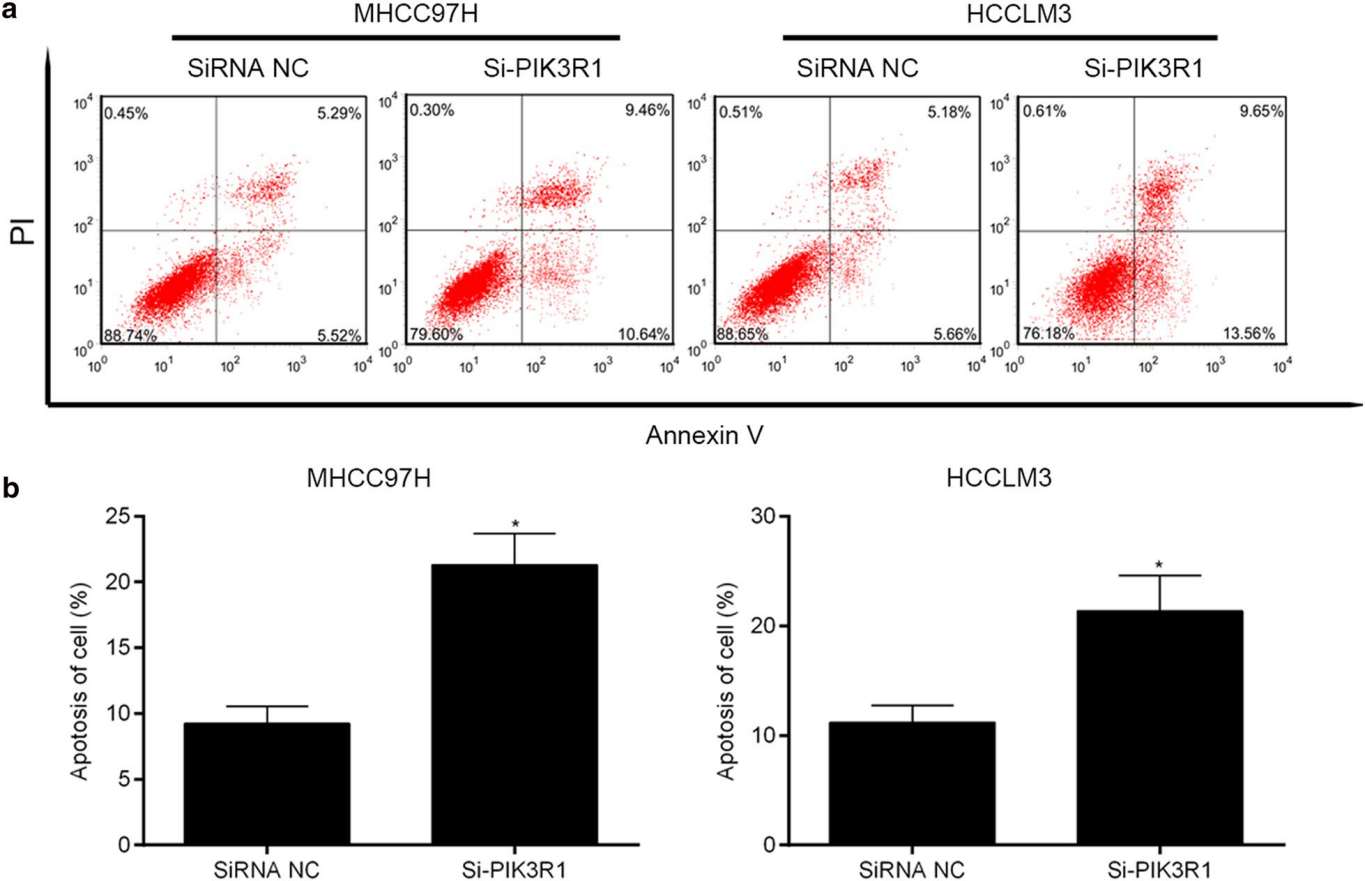
Down-regulation of PIK3R1 expression by siRNA increases apoptosis in MHCC97H and HCCLM3 cells. **a** Flow cytometry detection of cell apoptosis; **b** quantification of apoptosis

Additionally, flow cytometry was used to examine cell apoptosis. Compared with control group, the apoptotic rate of MHCC97H-si PIK3R1 and HCCLM3-si PIK3R1 cells were significantly increased (Fig. 5a, b). Thus, the down-regulation of PIK3R1 expression by siRNA increases apoptosis in HCCLM3 and MHCC97H cells.

### Knockdown of PIK3R1 downregulated p-PI3K, p-AKT, and p-mTOR expressions in MHCC97H and HCCLM3 cells

**Fig. 6:**
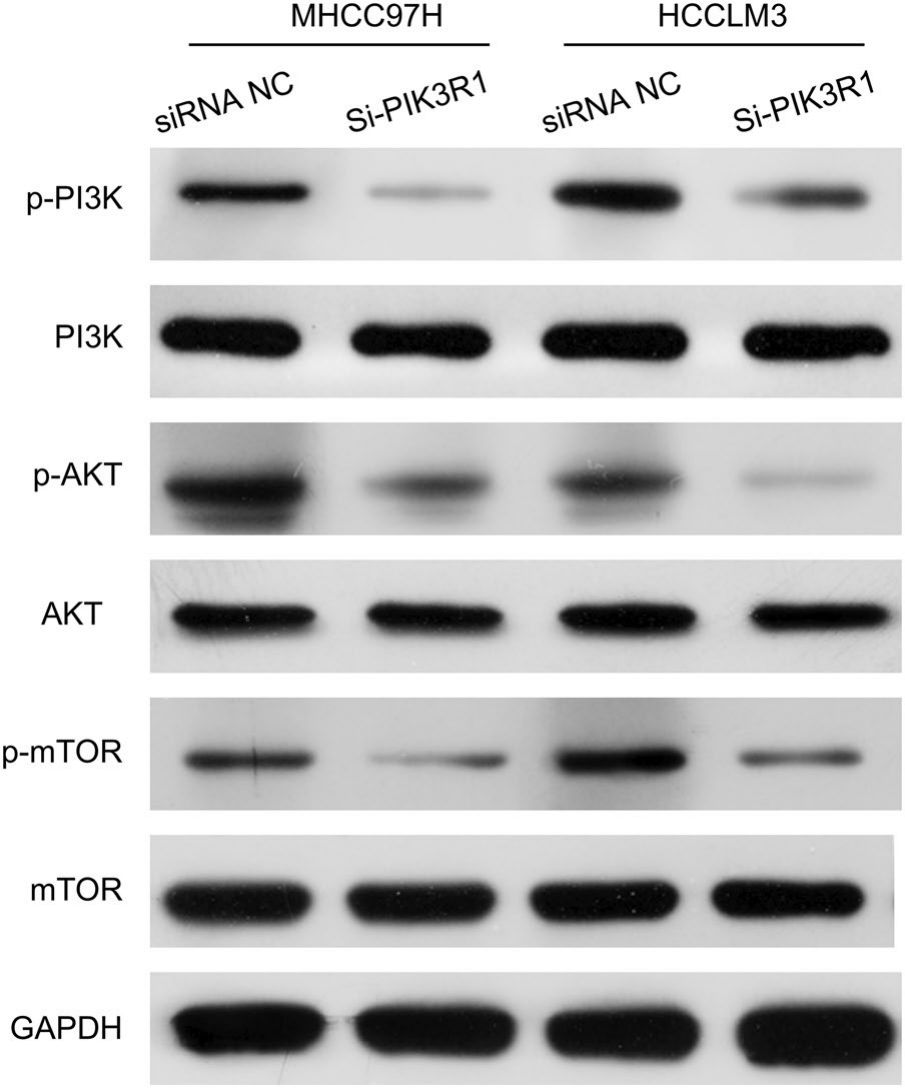
Knockdown of PIK3R1 downregulated p-PI3K, p-AKT, and p-mTOR in MHCC97H and HCCLM3 cells. The protein expression levels of PI3K, p-PI3K, AKT, p-AKT, mTOR and p-mTOR were assessed by Western blot assay in MHCC97H and HCCLM3 cells after PIK3R1 knockdown. GAPDH was used as loading control

In order to investigate the possible mechanism of PIK3R1 in HCC, MHCC97H and HCCLM3 cells were transfected with PIK3R1 siRNAs, respectively. The results showed that the protein expression levels of p-PI3K, p-AKT, and p-mTOR were downregulated in si-PIK3R1 group compared with siRNA NC group. These data demonstrated that knockdown of PIK3R1 by siRNAs inhibited p-PI3K, p-AKT, and p-mTOR expressions in MHCC97H and HCCLM3 cells (Fig. 6).

## Discussion

PIK3R1 has been shown to play important roles in many developmental processes, including cancer. In recent years, researchers found that PIK3R1 was abnormally expressed in various tumors is related to increased cell proliferation and invasion and reduced apoptosis [10]. Although PIK3R1 has been proved to function as an oncogene in many malignances, the relationship between PIK3R1 and HCC has not been fully elucidated. In the present study, we employed immunohistochemistry, qRT-PCT and western blot to accurately detect the expression level of PIK3R1 in HCC tissues. Besides, we used two HCC cell lines to explore the possible regulatory mechanism of PIK3R1 in the tumorigenesis of HCC. We described here that both the protein and mRNA levels of PIK3R1 was highly expressed in most human primary HCC tissues, whereas lowly expressed in adjacent normal liver tissues, suggesting the important roles of PIK3R1 in human HCC tumorigenesis.

Cancer has biological characteristics including abnormal cell differentiation and proliferation, uncontrolled growth, infiltration and metastasis. The occurrence of cancer is a complex process with multiple factors and steps, which can be divided into three processes: carcinogenic, tumor promotion and progressive [11, 12]. In addition, apoptosis, serves as a crucial part of biological process, is a gene-controlled cell-independent death process, and a vital mechanism to maintain stable internal environment [13]. In our study, we proved that PIK3R1 expression was significantly increased in our six HCC cell lines, especially in HCCLM3 and MHCC97H cells, which exhibited the much higher metastatic ability, revealing that PIK3R1 may promote the tumor metastasis. Therefore, we selected HCCLM3 and MHCC97H cells to further investigate the involvement of PIK3R1 in HCC progression. We had carefully evaluated the direct effect of PIK3R1 on the ability of cell proliferation, apoptosis and migration. Inhibition of PIK3R1 was found to suppress the proliferation and colony forming capability of HCCLM3 and MHCC97H cells compared with control-siRNA cells. In addition, we demonstrated that knockdown of PIK3R1 inhibited the migration and promoted apoptosis of HCC cell lines. Combining with the previous reports, these observations further confirmed the oncogenic roles of PIK3R1 in HCC. The downregulation of PIK3R1 led to growth inhibition of HCC cells, which might be correlated with cell arrest in G2/M phase of cell cycle and apoptosis enhancement [14, 15]. As we know, PI3K is a dimeric enzyme consisting of a catalytic (p110) and a regulatory subunit (p85α). p85α, encoded by PIK3R1, is reported to be an oncogene in ovarian, colorectal and prostate cancers, so exploring the role of p85α in HCC may provide unique insights into activation of PI3K/AKT pathway [16, 17]. Previous researches had revealed that PIK3R1 silencing could repress Huh7 proliferation, which is consistent with the other research that the deletion or reduction of PIK3R1 impaired B cell development and proliferation, delayed embryonic body development and inhibited cell adhesion [18, 19]. In addition, studies showed that PIK3R1 participated in the epithelial-mesenchymal transition of renal cancer cells [7]; PIK3R1 played an essential role kidney cancer [20]; PIK3R1 was involved in the migration and invasion of breast cancer byPI3K/AKT signaling [21]. Thus, PIK3R1 might be a potential target for cancer therapy.

A great deal of researches that showed that phosphatidylinositol 3-kinase (PI3K) signaling pathway is closely related to the occurrence and development of various human tumors, including HCC [22, 23]. Protein kinase B (PKB/AKT), a serine/threonine (Ser/Thr) protein kinase, is the main effector of PI3K downstream [24]. The aberrant expression of AKT can be detected in multiple malignant tumor cells such as HCC [25–27]. The mechanistic target of rapamycin (mTOR), an atypical Ser/Thr protein kinase, is a downstream effector protein of AKT, which regulates transcription and protein synthesis, and has an important influence on growth and proliferation of tumor cells [28–30]. However, whether PIK3R1 can regulate the pI3K-AKT-mTOR signaling pathway in HCC cells has not been reported. In our study, we further demonstrated that knockdown of PIK3R1 obviously downregulated p-PI3K, p-AKT, and p-mTOR expressions in HCC cells, suggesting that knockdown of PIK3R1 inhibited PI3K/AKT/mTOR pathway in HCC.

## Conclusions

In conclusion, we found that PIK3R1 expression was upregulated in the majority of HCC clinical tissue specimens, silence of PIK3R1 suppressed cell proliferation, migration, and accelerated apoptosis of HCC cells. In addition, silence of PIK3R1 decreased p-PI3K, p-AKT, and p-mTOR expressions in HCC. These findings provide information that will facilitate development of a novel therapeutic approach against HCC. However, further studies are needed to explore the possible mechanisms of PIK3R1 on HCC proliferation, apoptosis and migration. Moreover, it will be necessary to determine the deeper functions and mechanisms of PIK3R1 on HCC in vivo.

## Abbreviations

PIK3R1: phosphoinositide-3-kinase, regulatory subunit 1
HCC: hepatocellular carcinoma
PI3K: phosphoinositide 3-kinase
EC: endometrial cancers
DAB: diaminobenzidine
SD: standard deviation
